# Have We Forgotten Auditory Sensory Memory? Retention Intervals in Studies of Nonverbal Auditory Working Memory

**DOI:** 10.3389/fpsyg.2016.01892

**Published:** 2016-12-02

**Authors:** Michael A. Nees

**Affiliations:** Department of Psychology, Lafayette College, EastonPA, USA

**Keywords:** auditory sensory memory, working memory, auditory cognition, nonverbal sounds, music cognition

## Abstract

Researchers have shown increased interest in mechanisms of working memory for nonverbal sounds such as music and environmental sounds. These studies often have used two-stimulus comparison tasks: two sounds separated by a brief retention interval (often 3–5 s) are compared, and a “same” or “different” judgment is recorded. Researchers seem to have assumed that sensory memory has a negligible impact on performance in auditory two-stimulus comparison tasks. This assumption is examined in detail in this comment. According to seminal texts and recent research reports, sensory memory persists in parallel with working memory for a period of time following hearing a stimulus and can influence behavioral responses on memory tasks. Unlike verbal working memory studies that use serial recall tasks, research paradigms for exploring nonverbal working memory—especially two-stimulus comparison tasks—may not be differentiating working memory from sensory memory processes in analyses of behavioral responses, because retention interval durations have not excluded the possibility that the sensory memory trace drives task performance. This conflation of different constructs may be one contributor to discrepant research findings and the resulting proliferation of theoretical conjectures regarding mechanisms of working memory for nonverbal sounds.

Atkinson and Shiffrin’s (1968) influential memory model described a *sensory register*, a *short-term store*, and a *long-term store*. Neisser (1967) dubbed the auditory sensory store *echoic memory*—a term synonymous with *auditory sensory memory* (ASM). Contemporary research has established that ASM is: (1) auditory modality-specific; (2) high in resolution, which seems to indicate storage of episodic rather than categorical or abstract information; (3) limited in duration; and (4) independent from attentional processes (Näätänen et al., 1989; Winkler and Cowan, 2005). Thus, ASM is a passive store of just-heard sounds that retains a “synthesized auditory memory” (Massaro, 1975)—a set of acoustic features organized in time that can be consulted to complete behavioral tasks, including comparing sounds to one another (e.g., Crowder, 1982).

Auditory sensory memory is qualitatively different from post-sensory memory processes (Massaro, 1972; Crowder, 1982), including Atkinson and Shiffrin’s short-term store, which evolved into the *working memory* (WM) construct (see, e.g., Logie and Cowan, 2015). WM receives input from sensory memory about recent perceptual experiences, maintains and manipulates information during in-progress cognitive activities, and interfaces with long-term memory to reinstate information from the latent, permanent corpus of previous experiences. These “working” aspects of WM— active mental manipulation, rehearsal, and reinstatement of information— represent an important functional distinction between WM and ASM. ASM does not involve active manipulation or rehearsal (see, especially, Green and McKeown, 2007, Experiment 2; also see McKeown and Mercer, 2012) and is insensitive to attentional processes (Näätänen et al., 1989; Winkler and Cowan, 2005), including WM rehearsal processes (Nees and Best, 2013; Nees and Walker, 2013). Further, ASM is only engaged when a sound is heard, whereas WM for sounds can be initiated in the absence of hearing a stimulus (i.e., when a sound is reinstated from long-term memory, as in auditory imagery).

## ASM in Verbal Versus Nonverbal Auditory WM Tasks

Contributions of ASM to task performance have been acknowledged in studies of WM for speech and language (i.e., verbal WM). Auditory verbal WM often has been studied using serial recall tasks. Participants hear a list of words, letters, or digits, then immediately write or speak aloud all of the items. Analysis of memory for each list position permits dissociations of performance driven by WM rehearsal processes (e.g., primacy effects—recall advantages in the early portion of lists due to extended rehearsal time) from performance driven by ASM (e.g., auditory recency—better recall for the last few items with auditory presentation). Thus, in the verbal WM literature, the respective contributions of WM rehearsal and ASM have been disentangled in empirical investigations and their accompanying theoretical interpretations (see Jones et al., 2004, 2006).

Nonverbal sounds typically are not amenable to recall tasks, because participants would need to respond verbally (e.g., by labeling the sound, see Paivio et al., 1975; also see Kumar et al., 2013 for an exception). Thus, participants might rehearse the sounds in WM as their verbal labels rather than remembering the sounds *per se*. Instead of recall tasks, studies of nonverbal auditory WM often have used two-stimulus comparison tasks (for a review, see Cowan, 1984). Participants hear an initial sound and compare it to a second sound following a retention interval. The duration of the retention interval—the time during which the initial sound must be remembered—typically has been a few seconds. For example, Deutsch’s seminal studies of tonal memory (reviewed in Deutsch, 1975) used 5 s retention intervals. Intervals of a few seconds became conventional for several reasons. Memory performance declines over time (Cowan et al., 1999), so the interval must be short enough to leave some memory intact over the short-term. To capture post-sensory processes, however, retention intervals must be long enough to exceed the duration of ASM. Researchers initially speculated that the maximum duration of the ASM trace was about 2 s or less (e.g., Neisser, 1967; Crowder, 1976), which suggested that negligible contributions of ASM to memory performance could be assumed when intervals exceeded 2 s. Further, practical considerations when implementing experimental procedures (e.g., participant fatigue or methods requiring a large number of trials) make shorter retention intervals attractive.

## ASM and WM Occur in Parallel

The retention interval must extend beyond the persistence of ASM to isolate WM processes, because information about the most recently heard stimulus is simultaneously available to both ASM and WM (e.g., rehearsal) processes. Deutsch (1975, p. 110) noted “…we can store nonverbal stimulus attributes over substantially longer time periods…It must be concluded that the sensory attributes of a stimulus survive in memory after verbal encoding, and that they continue to be retained in parallel with the verbal attributes.” Similar views on parallel access to ASM and WM were expressed in seminal memory texts (Massaro, 1975; Crowder, 1976; Underwood, 1976).

Empirical findings have supported parallel representation in ASM and WM. Nees and Walker (2013) asked participants to encode two-note sound sounds with increasing or decreasing intervals, visual words (“increasing” or “decreasing”), or images (simple increasing or decreasing graphs). All stimulus forms indicated equivalent information: either an increasing or decreasing state. WM encoding strategies also were manipulated; participants were instructed to encode and rehearse the initial stimulus as either a sound (i.e., auditory imagery), a word (verbal encoding), or an image (visual imagery). Following a 3 s retention interval, participants made a speeded same/different response to a second stimulus, which could be either a sound, a word, or an image. Response times to the second stimulus were examined across factorial combinations of initial stimulus format, encoding strategy, and response stimulus format. Results generally showed that participants responded faster when the format of the second stimulus matched the strategy with which they rehearsed the initial stimulus in WM, but results also showed an independent effect of the initial stimulus format. When the initial stimulus was a sound, participants were faster to respond when the second stimulus was also a sound, regardless of (i.e., collapsed across) the WM encoding strategy. No such compatibility between the stimulus formats was observed for the visually presented words or images. These findings demonstrated that the effects of ASM persisted in parallel with recoding in WM (also see Nees and Best, 2013).

**FIGURE 1:**
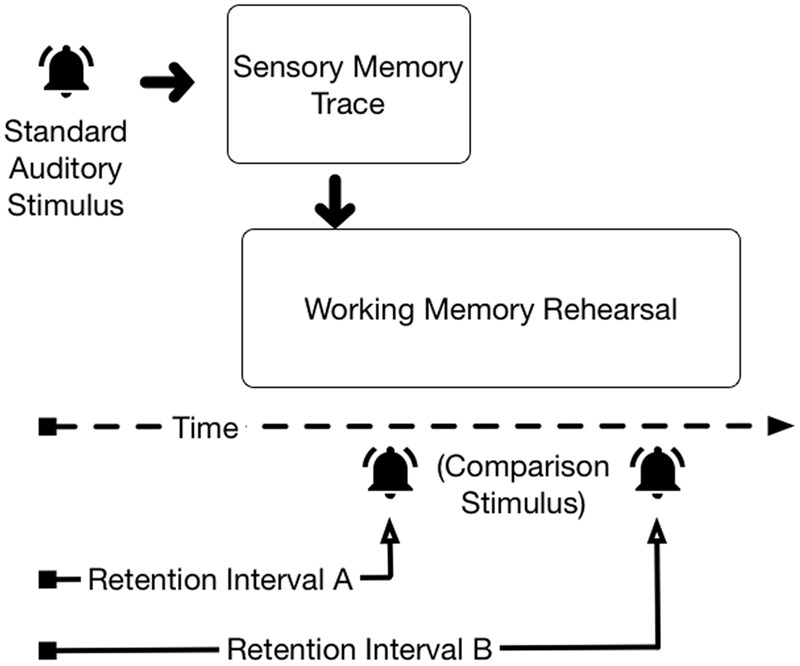
**Hypothetical availability of the ASM trace for two different retention intervals**.

Simultaneous representation in ASM and WM presents difficulties for isolating the construct of interest during performance of two-stimulus comparison tasks. **Figure 1** depicts two retention intervals following the offset of an auditory stimulus in a two-stimulus comparison task. With Retention Interval A, the participant could consult either the lingering ASM trace^1^ or the rehearsed WM trace to decide whether the standard and comparison stimuli are the same or different; both memory traces exist in parallel. With retention Interval B, ASM is no longer available when the comparison stimulus arrives; performance of the task must be accomplished using the rehearsed WM representation of the standard stimulus. Interpretation of observed memory performance for Retention Interval A is ambiguous—participants’ memory performance could reflect the fidelity of the ASM trace, the fidelity of the representation rehearsed in WM, or some combination of information from both sources.

As such, the attribution of task performance to WM mechanisms in two-stimulus comparison tasks hinges on the assumption that the ASM trace does not survive the duration of the retention interval. This assumption may be questionable for brief retention intervals. Research has shown striking variation in the estimated duration of ASM. Though some researchers have estimated it to be 2 s or less (Crowder, 1976; Huron and Parncutt, 1993), longer estimates have included 3.5 s (Mcevoy et al., 1997), 4–5 s (Glucksberg and Cowen, 1970), at least several seconds (Cowan, 1984), 10 s (Sams et al., 1993), 10–15 s (Winkler and Cowan, 2005), 20 s (Watkins and Todres, 1980), at least 30 s (Winkler et al., 2002), and possibly up to 60 s (Engle and Roberts, 1982).

## ASM and WM in Cognitive Neuroscience

Cognitive neuroscience research has corroborated parallel ASM and WM processes (e.g., Buchsbaum et al., 2005), and separate neurological markers have been identified for ASM and auditory WM processes. The widely researched mismatch negativity (MMN) component (see Näätänen, 2000) of evoked neural responses to sounds offers a metric of the duration of ASM. In a review of MMN studies, Schröger (2007) concluded that ASM may endure up to 20 s or longer. Regarding auditory WM, recent research (Lim et al., 2015) showed that processes involving maintenance of sounds are indexed by oscillations that fall within the alpha range of frequencies in electroencephalography (EEG) recordings. Wilsch and Obleser (2016) suggested that the power fluctuations of alpha oscillations track top-down attentional processes that serve to maintain representations in WM, perhaps while simultaneously inhibiting incoming sensory input that could potentially interfere with maintenance (also see Zimmermann et al., 2016).

## Forgetting Sensory Memory?

Despite evidence that ASM is distinct from WM and may persist for longer than a couple of seconds, results from two-stimulus comparison tasks in studies of nonverbal auditory WM have been interpreted with indifference toward ASM. In recent research reports on nonverbal auditory stimuli, the term “WM” has been used to describe memory over *any period of time whatsoever* following hearing a sound. Golubock and Janata (2013) defined retention of a sound for as brief as 1 s following stimulation as a WM task. Using retention intervals as brief as 3 s, Soemer and Saito (2015) likewise implied that the retention of a sound for any duration following stimulation must be accomplished by an active WM maintenance mechanism (also see Schulze et al., 2012). Schendel and Palmer (2007) used a 4.2 s retention interval in their study of mechanisms of WM for melodies. Li et al. (2013) and Siedenburg and McAdams (2016) used 6 s retention intervals in studies of nonverbal auditory WM. Though sounds may indeed engage WM processes immediately following perception, parallel access to ASM also may have influenced task performance in some studies that attempted to examined WM processes.

Is this apparent oversight semantic or substantive? Some researchers may have equated WM with “short-term” memory in the most literal sense (i.e., without intending to differentiate ASM and active WM rehearsal), as memory terminology has been used ambiguously in the literature (see Cowan, 2008). Yet research procedures that purport to examine active mechanisms of rehearsal and maintenance in nonverbal WM seem to face a substantive interpretive challenge when the contributions of ASM are overlooked. With brief retention intervals, some memory tasks may be accomplished using ASM—a different construct from WM altogether.

This ambiguity is especially problematic when interfering tasks or stimuli are introduced during the retention interval of two-stimulus comparison tasks to infer mechanisms of active rehearsal in WM. According to the logic of these paradigms, a secondary task that requires the same WM mechanism as rehearsal of the sound will reduce memory performance (see Heuer, 1996). Lack of interference indicates the mechanism of the secondary task is not involved in WM for the sound stimuli. As a representative example, articulatory suppression (i.e., repeating an irrelevant word or syllable) has been used during retention intervals to examine the extent to which articulation is involved in rehearsal of nonverbal sounds. In a recent application of this paradigm by Soemer and Saito (2015), participants heard two, three, or four abstract sounds (discriminable by timbre) followed by a retention interval (either 3 s or 12 s). Participants then indicated if a single probe was one of the initial sounds. Articulatory suppression (repeating “da” aloud) was used during the retention interval. Results showed no effects of articulatory suppression compared to a control condition, except for two-item lists^2^. Using a similar procedure with a retention interval of 6 s, however, Siedenburg and McAdams (2016) reported that articulatory suppression did impair memory performance compared to a control condition. Both studies attempted to draw conclusions about mechanisms of active maintenance in WM. Since we do not have a precise estimate of the duration of ASM, performance arguably could have reflected ASM, WM rehearsal, or some combination of both, especially with a 3 s retention interval and perhaps even with a 6 s interval. Even when the WM rehearsal mechanism for a stimulus has been blocked, task performance may remain partially or even fully intact due to contributions from ASM (e.g., McKeown et al., 2011). In this case, an observed lack of interference could erroneously suggest that the interference task did not require the same WM mechanism as retention of the stimulus.

## Advancing Theories of Nonverbal Auditory WM

Discrepant findings like those discussed above have led to a range of theoretical perspectives on the active processing (e.g., rehearsal) of nonverbal sounds in WM. Researchers have suggested WM for nonverbal sounds is accomplished by: (1) the phonological loop of verbal WM (Baddeley and Logie, 1992); (2) an independent “music memory loop” (Berz, 1995); (3) attention (Siedenburg and McAdams, 2016); and (4) different mechanisms for pitch versus timbre (Soemer and Saito, 2015). Further, some (e.g., Demany and Semal, 2008) have even suggested that rehearsal of nonverbal auditory stimuli is not possible. These disparate proposals indicate an area in need of more research that focuses intensively on theory-building. A successful theory of the mechanism of WM for nonverbal sounds will need to differentiate ASM from WM.

To reveal the properties of a WM rehearsal mechanism for nonverbal sounds, ASM’s effects should be minimized in studies that purport to assess rehearsal. The indeterminate duration of ASM precludes recommending a definitive retention interval duration that would eliminate contributions of ASM. To complicate the matter further, evidence has suggested that the duration of ASM is subject to considerable individual differences (e.g., Kubovy and Howard, 1976). Clearly, longer retention intervals will be less likely to allow for ASM to contribute to performance on tasks for which the target construct is WM. Intervals of less than 5 s are well within the persistence of many estimates of the behavioral life of ASM, whereas intervals of 8–10 s or more begin to exceed the duration of many, but not all, estimates of the duration of ASM.

Elimination of the ASM trace with irrelevant auditory stimuli may offer another solution. Some researchers have appended an irrelevant sound following the presentation of to-be-remembered sounds in an attempt to overwrite the ASM trace. Soemer and Saito used a 200 ms burst of white noise following their to-be-remembered stimuli. Li et al. (2013) used a 500 ms composite sound—all 12 test tones in their experiment presented concurrently. Although this approach makes sense intuitively, care must be taken to ensure that these post-stimulus masks actually overwrite ASM. The auditory version of the suffix effect—whereby memory for the last few items in auditory serial lists is impaired by presentation of a post-list sound—has been taken to reflect interference in ASM (for a detailed review, see Penney, 1989)^3^. The suffix effect has been studied extensively in verbal WM, and some studies have examined the effect for nonverbal auditory stimuli. Interference by a suffix depends upon the acoustic similarity between the suffix and its preceding stimulus (e.g., Morton et al., 1971; Rowe and Rowe, 1976). Greene and Samuel (1986) showed that white noise did not result in a suffix effect for verbal digits or non-speech tones, which casts doubt on the effectiveness of white noise as a stimulus that overwrites ASM. Interestingly, they also showed that a speech suffix and a non-speech chord suffix both seemed to overwrite ASM for tones, but only speech showed a suffix effect for digits. Overwriting in ASM requires more research, but without definitive empirical evidence, it seems unwarranted to assume that a noise burst or a composite stimulus will eliminate contributions of ASM to WM tasks.

Attempts to develop a response modality that permits recall (rather than recognition) tasks with nonverbal auditory stimuli also could be useful. As has been the case with verbal WM, recall of lists of nonverbal auditory stimuli could perhaps reveal patterns of serial position errors that differentiate WM rehearsal from ASM. The difficulty is that common response modalities create task demands that encourage participants to translate sounds into a different memory code, which may defeat the goal of examining memory for sounds *per se*. Researchers have devised creative approaches to nonverbal auditory memory tasks to circumvent this problem (e.g., Kumar et al., 2013), but more research is needed on this topic.

Finally, cognitive neuroscience approaches could help to clarify the relative contributions of ASM and WM to performance of auditory memory tasks. Research has suggested that behavioral performance that correlates with the MMN would likely reflect memory contributions from ASM (Näätänen, 2000; Schröger, 2007), whereas performance that correlates with alpha oscillations would reflect contributions from maintenance processes in WM (see Lim et al., 2015).

## Conclusion

Discrepant findings have hindered the development of theory regarding mechanisms of rehearsal in nonverbal auditory WM. Procedures that conflate ASM with WM may be one potential contributor to disparate results. Task demands established by methodological decisions about the duration of the retention interval in two-stimulus comparison tasks may have allowed for ASM to affect results that have been attributed to rehearsal in WM. A viable theory of nonverbal auditory WM will need to explain the relationship between ASM and WM. A renewed focus on this relationship and the potential role of ASM in performance of WM tasks may be valuable in this regard.

## Author Contributions

The author confirms being the sole contributor of this work and approved it for publication.

## Conflict of Interest Statement

The author declares that the research was conducted in the absence of any commercial or financial relationships that could be construed as a potential conflict of interest.

## Footnotes

1 For simplicity, ASM is shown terminating discretely in **Figure 1**. Research has shown the sensory trace has a half-life (e.g., Kubovy and Howard, 1976), which suggests that the trace decays as a function of time (see, e.g., McKeown and Mercer, 2012).

2 Their graphical results (their Figure 1, upper right) suggested a trend toward an interaction such that suppression had a detrimental effect at 12 s but not 3 s, but the statistical analysis of that interaction was not reported.

3 Penney (1989, pp. 403–404) viewed suffix effects as a family of short-term memory phenomena, with the *auditory version* attributable to ASM.
